# Characterisation of the wheat (*triticum aestivum* L*.*) transcriptome by *de novo* assembly for the discovery of phosphate starvation-responsive genes: gene expression in Pi-stressed wheat

**DOI:** 10.1186/1471-2164-14-77

**Published:** 2013-02-04

**Authors:** Youko Oono, Fuminori Kobayashi, Yoshihiro Kawahara, Takayuki Yazawa, Hirokazu Handa, Takeshi Itoh, Takashi Matsumoto

**Affiliations:** 1 Agrogenomics Research Center, National Institute of Agrobiological Sciences, 2-1-2 Kannondai, Tsukuba, Ibaraki, 305-8602, Japan; 2 Government and Public Corporation Information Systems, Hitachi Co., Ltd., 2-4-18 Toyo, Koto-ku, Tokyo, 135-8633, Japan

**Keywords:** *De novo* assembly, RNA-Seq, Transcriptome, Wheat, Phosphorus, Phosphate starvation

## Abstract

**Background:**

Phosphorus (P) is an essential macronutrient for plant growth and development. To modulate their P homeostasis, plants must balance P uptake, mobilisation, and partitioning to various organs. Despite the worldwide importance of wheat as a cultivated food crop, molecular mechanisms associated with phosphate (Pi) starvation in wheat remain unclear. To elucidate these mechanisms, we used RNA-Seq methods to generate transcriptome profiles of the wheat variety ‘Chinese Spring’ responding to 10 days of Pi starvation.

**Results:**

We carried out *de novo* assembly on 73.8 million high-quality reads generated from RNA-Seq libraries. We then constructed a transcript dataset containing 29,617 non-redundant wheat transcripts, comprising 15,047 contigs and 14,570 non-redundant full-length cDNAs from the TriFLDB database. When compared with barley full-length cDNAs, 10,656 of the 15,047 contigs were unalignable, suggesting that many might be distinct from barley transcripts. The average expression level of the contigs was lower than that of the known cDNAs, implying that these contigs included transcripts that were rarely represented in the full-length cDNA library. Within the non-redundant transcript set, we identified 892–2,833 responsive transcripts in roots and shoots, corresponding on average to 23.4% of the contigs not covered by cDNAs in TriFLDB under Pi starvation. The relative expression level of the wheat *IPS1* (*Induced by Phosphate Starvation 1*) homologue, *TaIPS1*, was 341-fold higher in roots and 13-fold higher in shoots; this finding was further confirmed by qRT-PCR analysis. A comparative analysis of the wheat- and rice-responsive transcripts for orthologous genes under Pi-starvation revealed commonly upregulated transcripts, most of which appeared to be involved in a general response to Pi starvation, namely, an IPS1-mediated signalling cascade and its downstream functions such as Pi remobilisation, Pi uptake, and changes in Pi metabolism.

**Conclusions:**

Our transcriptome profiles demonstrated the impact of Pi starvation on global gene expression in wheat. This study revealed that enhancement of the Pi-mediated signalling cascade using *IPS1* is a potent adaptation mechanism to Pi starvation that is conserved in both wheat and rice and validated the effectiveness of using short-read next-generation sequencing data for wheat transcriptome analysis in the absence of reference genome information.

## Background

As a key component of plant cell molecules, phosphorus (P) is an essential macronutrient for plant growth. Large quantities are used in fertiliser, but worldwide P resources will be exhausted by the end of this century [1]. Phosphate (Pi) starvation can generally be observed throughout an afflicted field. Visual symptoms of Pi starvation (−P) are the development of dark-green leaf colour and a reduction in shoot elongation and leaf size. As −P progresses in wheat (*Triticum aestivum* L.), the oldest leaves become chlorotic and show signs of desiccation [2].

Wheat is a major staple food crop in many parts of the world in terms of both cultivation area and prevalence as a food source. To meet the increasing global demand for wheat, this crop’s exploitation of nutrients must be made more efficient and its requirement for nutritional fertilisers reduced. Because wheat is primarily grown on substrates with low P levels, such as the acidic soils of tropical and subtropical regions and the calcareous soils of temperate regions, an important constraint to wheat production is its lack of tolerance to −P.

Various genetic approaches have been used to understand genetic control of −P tolerance in wheat; these include aneuploid analyses of the nulli-tetrasomic series and wheat alien chromosome addition lines of the cultivar ‘Chinese Spring’ (CS) and quantitative trait locus (QTL) mapping [3-6]. QTL analyses using –P-sensitive CS and the tolerant variety ‘Lovrin 10’ indicated that CS possesses positive alleles of the major QTLs for P use efficiency on chromosomes 3B, 4B, and 5A [4]. In another study, seven and six QTLs were repeatedly detected controlling P uptake and use efficiency [5]. A large number of QTLs for agronomic trait changes under low or high P concentrations have been detected on all chromosomes in the hexaploid wheat genome, implying that −P tolerance is controlled by polygenes [5]. However, the studies are few in number; a reverse genetic approach could help characterise genes that potentially contribute to complex multilocus traits and their global transcriptional networks in Pi-starved wheat.

Several technologies, including massively parallel sequencing and microarray analysis, have recently been used to simultaneously catalogue the effects of −P on the expressions of thousands of genes in model species [7-10]. Transcriptome sequencing using next generation sequencing (NGS) technology provides high-resolution data and is a powerful tool for studying global transcriptional networks. The evaluation of sequence-based expression profiles can identify responsive genes and provide functional annotation for genes underlying complex and multilocus traits under −P in wheat.

In model species, transcriptome profiling and the quantification of gene expression levels are generally performed by mapping reads from the NGS analysis to a reference genome sequence and annotating genes. The strategies for model species are not feasible in wheat, as its reference genome sequence and gene annotation are still incomplete; an international project to achieve these goals is currently making progress (IWGSC: International Wheat Genome Sequencing Consortium, http://www.wheatgenome.org/). This project may take considerable time, because of the difficulties involved in sequencing the huge (40 times larger than rice), highly-repetitive hexaploid genome of wheat.

The database of putative full-length cDNAs for wheat, TriFLDB, has released approximately 16,000 full-length cDNAs (http://trifldb.psc.riken.jp/index.pl) [11]. Although this dataset is a useful reference for transcript mapping, it is incomplete, because 36,000 to 50,000 genes have been estimated per diploid genome based on the 3B chromosome of hexaploid wheat [12,13]. Recently, *de novo* transcript assembly analysis has made possible comprehensive analyses of transcriptomes, and several studies have detailed the transcriptome sequencing of various non-model species, including wheat, using massively parallel sequencing technology [14,15]. *De novo* assembly of short sequences of transcripts enables researchers to reconstruct the sequences of entire transcriptomes, identify and catalogue all of the expressed genes, separate isoforms, and capture transcript expression levels.

Although computer-based *de novo* assembly tools (e.g., Trans-ABySS, Velvet-Oases, and Trinity) [16-18] have been developed in conjunction with massively parallel sequencing, their usefulness in transcriptome assembly is not yet well demonstrated, and improvements can still be achieved using recent advances in bioinformatics. Some studies have used short-read sequence data obtained with the Illumina sequencer for *de novo* assembly; others have used the relatively long-read sequence data obtained with the Roche 454 pyrosequencing system or have adopted a hybrid approach of both short and long reads. In addition, contig construction is greatly affected by sequence read quality (i.e., length) and quantity. Furthermore, the cDNA library construction methods, sequencing technologies, and data pre-treatment techniques chosen influence the quality of the assembled transcriptomes [19]. Consequently, a comparison of several assembly programs is needed to determine the best combination of parameters, which can then serve as a guideline for sequence assembly performance.

In this study, we verified the *de novo* assembly approach by comparing analyses from several programs using short-read sequence data obtained from wheat cultivar CS seedlings under –P. We constructed a wheat transcript dataset for *de novo* assembly and quantified gene expression. As a reference in the gene expression analysis, we used a non-redundant set of transcripts generated from the *de novo* assembly and full-length cDNAs. This dataset was also used to assess transcripts, to investigate sequence similarity, for conservation analyses among several plant species, and for comparison with our previous report on rice transcript profiling under −P conditions [10]. We demonstrated that an overall mechanism regulating gene expression of −P-responsive genes in wheat could be effectively characterised using short-read NGS data. A comparison of gene expression profiles in wheat and rice revealed the presence of conserved gene expression systems, which appear to be essential to adaptation to –P conditions. Finally, we described an effective method for assembly of short transcript sequences to discover novel functional genes in the absence of a reference genome. The transcript assembly generated in this study should serve as a useful resource for wheat genomics and genetics.

## Results and discussion

### Construction of the wheat transcript dataset

**Figure 1:**
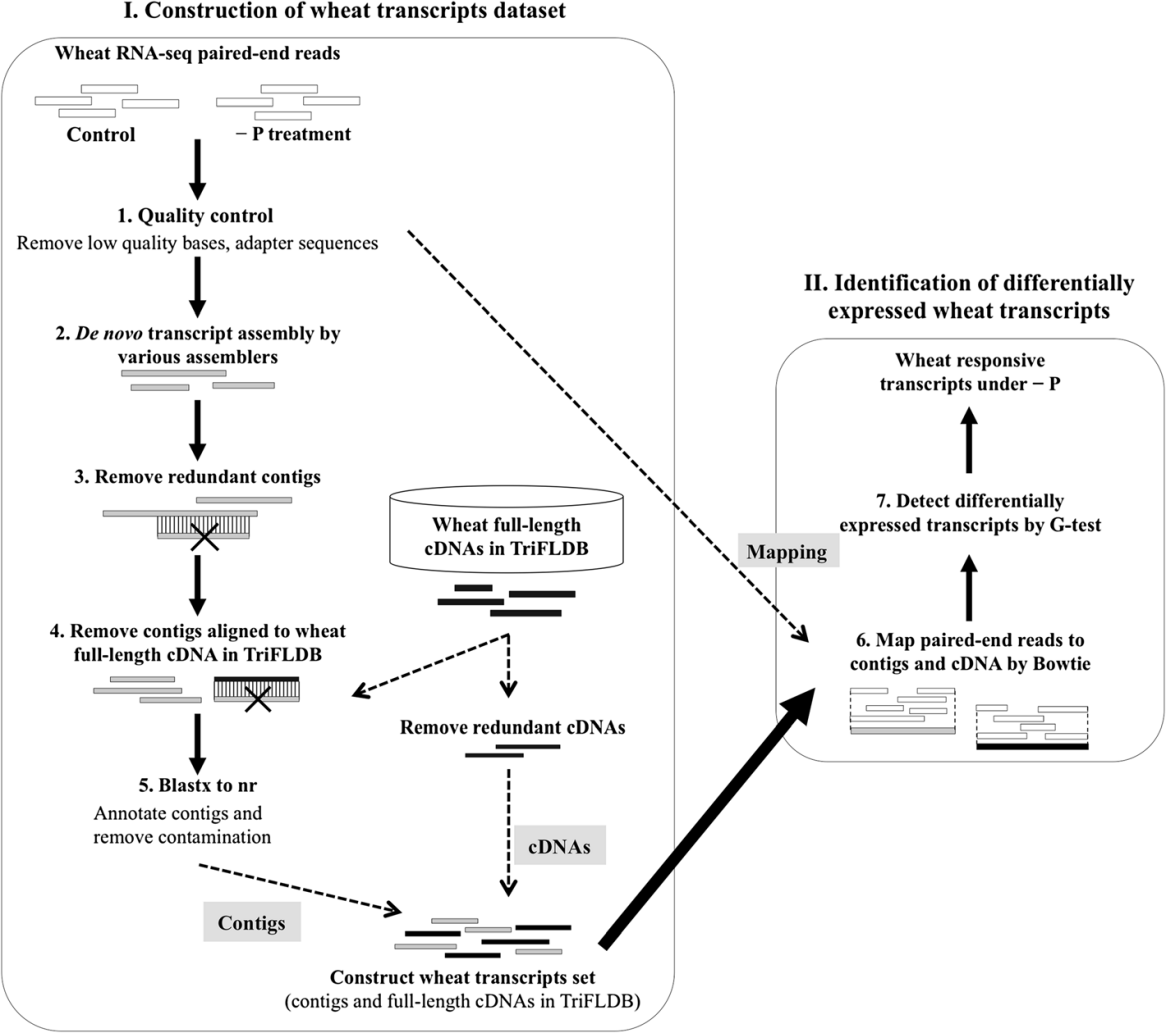
**Strategy used in the wheat RNA-Seq analysis.** A set of 29,617 wheat transcripts was constructed from 15,047 non-redundant contigs and 14,570 full-length cDNAs in TriFLDB. To identify the responsive transcripts under –P conditions, all reads were aligned to the transcripts using Bowtie, and a G-test with an FDR value cut-off < 0.01 was used.

A set of transcripts consisting of cDNAs from TriFLDB and contigs from RNA-Seq reads was constructed as shown in Figure 1 (steps 1–5). A total of 12 libraries (two tissues, two treatments, and three replicates) from seedlings subjected to –P (0 mM NaH_2_PO_4_; 0 mg L^−1^ P) and control (0.323 mM NaH_2_PO_4_; 10 mg L^−1^ P) treatments were used in the RNA-Seq analysis. Overall, 115 million paired-end short-read sequences were produced by an Illumina HighSeq 2000 system (Illumina, San Diego, CA, USA) and used for *de novo* assembly after the removal of low-quality segments. The choice of data pre-treatment technique, in which data are pre-processed to remove sequencing errors and other artefacts, influences the accuracy and precision of the gene expression analyses [19]. Trans-ABySS, Velvet-Oases (henceforth Oases), and Trinity were used for sequence assembly in this experiment (see Methods for details). Each contig included various transcript isoforms, which cause redundancy. Because their inclusion could present difficulties in a quantitative analysis, the redundant contigs were removed. The removal of these contigs generated a non-redundant set of contigs, which were assessed using various output parameters—such as number of contigs and mean contig length as a function of *k*-mer length—to select the appropriate assembly program. The largest assembly, comprising 555,287 contigs at least 101 bp long, was produced by Trinity, while the smallest, consisting of 337,969 contigs of at least 100 bp in length, was generated by Oases. However, Oases yielded the best results of the three assembly programs, with a maximum contig length of 22,610 bp, a mean contig length of 1,113 bp, and a median contig length of 698 bp (Additional file 1 and Additional file 2).

Contig quantity and mean length are often used in *de novo* assembly assessment, but these values may not reflect contig accuracy. We therefore assessed accuracy using the three assembly programs discussed above. We counted the number of contigs that could be aligned with 90% or greater coverage to their corresponding full-length cDNAs in TriFLDB (Additional file 3). Of the three assembly programs, Oases assembled the longest transcripts and had the highest percentage of alignments to the wheat full-length cDNAs. Although it generated the smallest number of contigs, Oases was able to reconstruct 87% of the TriFLDB cDNAs with identity ≥90% and coverage ≥50%, or 75% of the TriFLDB cDNAs with identity ≥90% and coverage ≥90% (Additional file 3). Conversely, Trinity produced the largest number of contigs, but could only reconstruct 16% of the TriFLDB cDNAs (identity ≥90%, coverage ≥90%). Trans-ABySS could reconstruct just 6% of the TriFLDB cDNAs (identity ≥90%, coverage ≥90%). Thus, the reconstruction percentages of the latter two programs were considerably worse than those of Oases, and we concluded that Oases was the most appropriate program for our analyses. Of the 337,969 contigs generated from Oases, 169,024 could not be aligned to the cDNAs using BLAT. When the longest contigs per locus were selected, 67,616 of them could not be aligned with the cDNAs.

Because the 67,616 non-redundant contigs could indicate homology to either plants or non-plants, we treated them as contaminants and removed them from the analysis, except when a BLASTX hit matched a land plant. The contigs were analysed for similarity/sequence conservation against nr (non-redundancy datasets of various species) using a BLASTX search (E-value ≤1E-03). A total of 15,047 contigs were found in land plants (top hit), with 91.4% in monocots, such as *Hordeum vulgare* ssp. *vulgare* (33.3%; 5,017 transcripts), *Brachypodium distachyon* (26.0%; 3,913 transcripts), *Oryza sativa* ssp. *japonica* group (13.5%; 2,025 transcripts), *Sorghum bicolor* (7.3%; 1,101 transcripts), *O. sativa* ssp. *indica* group (4.9%; 743 transcripts), *T. aestivum* (3.6%; 537 transcripts), *Zea mays* (2.1%; 317 transcripts), and other *H. vulgare* subspecies (0.7%; 100 transcripts) (Additional file 4). Notably, 10,656 of the 15,047 contigs could not be aligned to full-length cDNAs in the barley full-length cDNA database (http://barleyflc.dna.affrc.go.jp/hvdb/) using BLAT, suggesting that most of the contigs were distinct from the barley transcripts. Finally, we obtained 29,617 wheat transcripts by combining the 15,047 non-redundant contigs with 14,570 non-redundant full-length cDNAs (Steps 1–5 in Figure 1). This dataset is available upon request.

### Mapping reads to the wheat transcript dataset

Characterisations of contigs and cDNAs from the 29,617 transcripts are presented in Table 1. To determine whether our contigs reflected the effect of −P in wheat, 51,130,274 of 115,495,198 quality-controlled paired-end reads were aligned back to the wheat transcript dataset (contigs and cDNAs) using Bowtie (step 6 in Figure 1, Table 2), which is designed for short-read mapping onto the genome. We used 39.6–46.7% of the uniquely-mapped reads in the expression analysis of each treatment. Replicates in all treatments were highly correlated (coefficient > 0.94), and reads from the same treatment were merged to calculate reads per kilobase of exon model per million mapped reads (RPKM), which indicates the relative transcription amount. We observed that 70% (10,144) of the 14,570 non-redundant cDNAs were associated with at least one unique read. On average, 92% of transcript length was covered by reads, with coverage depth of each transcript averaging 420 reads. These statistics suggested that nearly complete coverage of the entire cDNA length was obtained in this analysis. Many reads were impossible to align back to the contigs, possibly because the redundant contigs had been removed in the previous analysis (Step 3 in Figure 1, with the longest contigs per locus selected by Oases).

**Table 1:** Characterisation of the 29,617 wheat transcripts obtained from contigs and cDNAs in TriFLDB

	**Contig**	**cDNA**
No. of transcripts	15,047	14,570
Maximum length	20,980	8,983
Minimum length	100	64
Average length	769	1,755
Median length	394	1,655
Transcripts larger than 1,000 bp	3,649	12,089

**Table 2:** Statistical summary of the reads aligned against the set of contigs and full-length cDNAs in TriFLDB

**Tissue**	**Total aligned**	**Unique-hit**	**%**	**Multi-hit**	**%**	**Unaligned**	**%**
Root_0d	21,505,572	8,793,897	40.9	2,166,997	10.1	10,544,678	49.0
Root_10d	20,619,073	8,161,939	39.6	1,971,013	9.6	10,486,121	50.9
Shoot_0d	37,116,104	17,335,224	46.7	6,676,313	18	13,104,567	35.3
Shoot_10d	36,254,449	16,839,214	46.4	6,046,477	16.7	13,368,758	36.9

TriFLDB: http://trifldb.psc.riken.jp/index.pl.

Distribution of RPKM values was assessed by comparing contigs and cDNAs (Additional file 5). Average RPKM values from each treatment ranged from 2.75–4.75 for contigs and 31.95–33.68 for cDNAs. The distributions of maximum and median RPKM values were lower for contigs than cDNAs, suggesting that most cDNAs were highly expressed, probably reflecting the overall distribution of gene expression in this study. We identified 529 contigs, however, with higher expressions than the average observed for cDNAs under one or more of the four tissue/treatment combinations. Some of these contigs may correspond to transcripts rarely represented in the full-length cDNA library or transcripts that were expressed at low levels in other conditions.

### Identification of differentially-expressed wheat transcripts under −P

**Figure 2:**
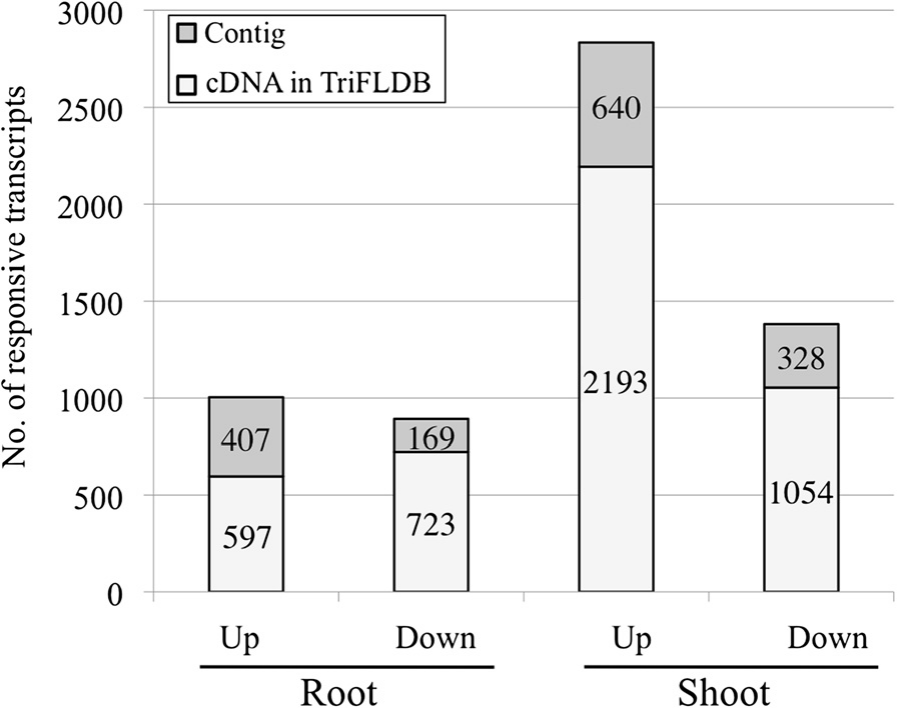
**Distribution of transcripts upregulated or downregulated in response to −P.** The total number of upregulated or downregulated transcripts identified by RNA-Seq was determined in roots and shoots under –P conditions. Each bar shows the distribution of transcripts with their matching cDNAs in TriFLDB (light grey) and contigs (grey).

A G-test (FDR [False Discovery Rate] <0.01) of the RPKM-derived read counts was performed to detect differences in gene expression between control and –P-treated plants, and to identify responsive wheat transcripts under –P conditions (step 7 in Figure 1). In roots, 1,004 transcripts were upregulated and 892 were downregulated; in shoots, 2,833 and 1,382 transcripts were upregulated and downregulated, respectively. The most numerous responsive transcripts were those upregulated in shoots, which were more than twice as abundant as those upregulated in roots (Figure 2). This result may be a consequence of the fact that most of the cDNAs in TriFLDB were constructed from shoot samples. On average, 23.4% of the transcripts in the responsive transcript set were contigs not covered by cDNAs in TriFLDB (Figure 2). The wheat transcript set contained more contigs than cDNAs, but there were more responsive cDNAs than contigs. The low-expression contigs might reflect small differences in expression between the two conditions that may not be detectable statistically, making the characterization of these transcripts difficult (Additional file 5). We were able to demonstrate, nonetheless, that the *de novo* assembly strategy can improve transcriptome analysis of a non-model species and that these upregulated or downregulated contigs could be functionally annotated as Pi responses in wheat under –P.

### Functional annotation of responsive transcripts under –P

**Figure 3:**
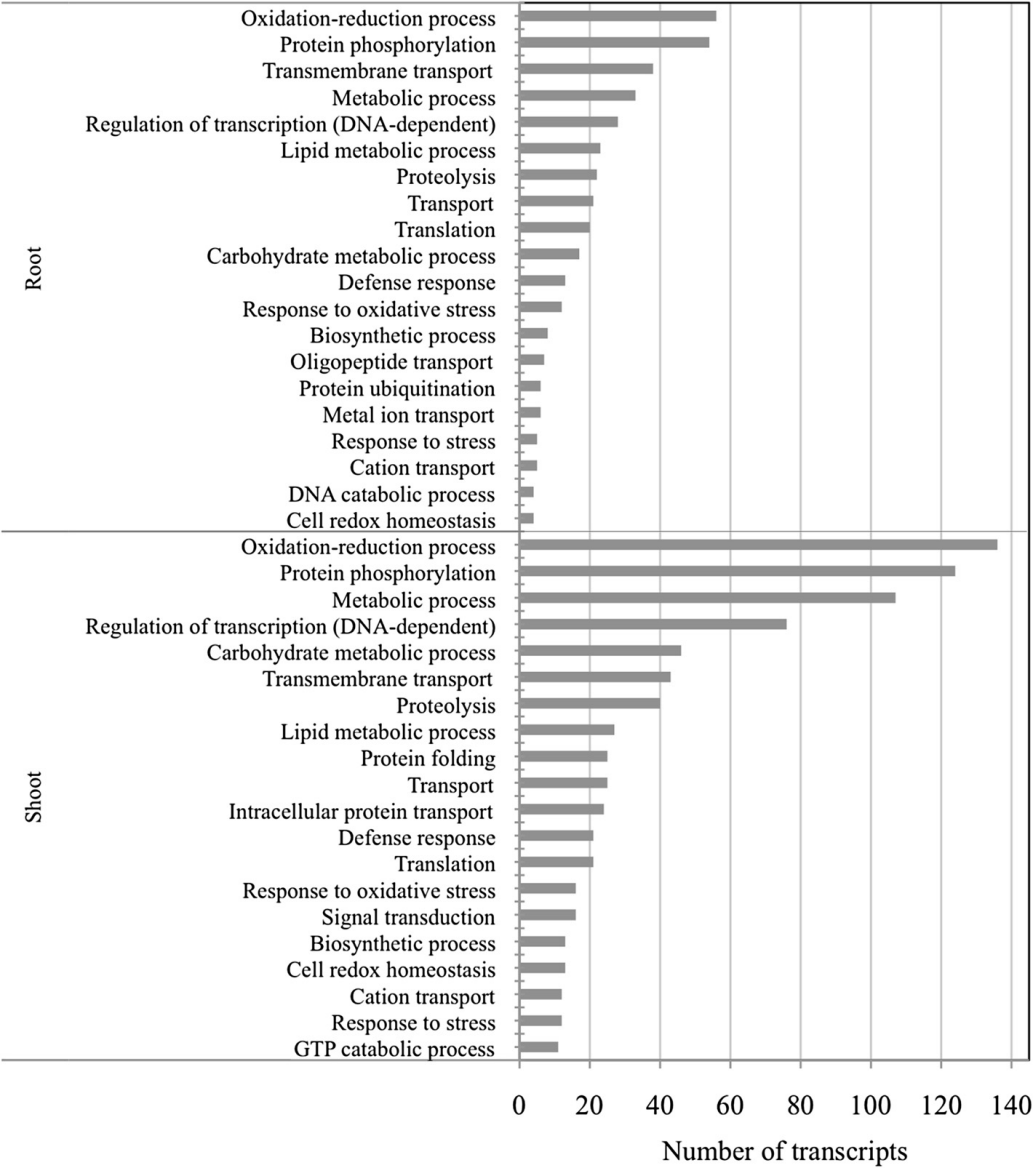
**Distribution of Gene Ontology (GO) biological process categories for upregulated contigs.** The number of upregulated transcripts in roots and shoots categorised in the top twenty GOs are summarised. The *y*-axis indicates the category. The *x*-axis indicates the number of transcripts in a category.

To obtain a functional annotation of upregulated wheat transcripts under –P conditions, we used Gene Ontology (GO) biological process categories. GO annotations were assigned to 40.7% (409) of the root transcripts and 34.4% (974) of the shoot transcripts. We identified the top 20 GO categories into which upregulated transcripts in roots and shoots were distributed (Figure 3). Although the numbers of transcripts differed, the overall categorisations were very similar between roots and shoots. Sixteen of the 20 GO categories were represented in both roots and shoots: oxidation-reduction process, protein phosphorylation, transmembrane transport, metabolic process, regulation of transcription (DNA-dependent), lipid metabolic process, proteolysis, transport, translation and carbohydrate metabolic process, defence response, response to oxidative stress, biosynthetic process, cation transport, response to stress, and cell redox homeostasis. Transcripts from both roots and shoots most belonged to the oxidation-reduction process category. This category was represented by many instances of cytochrome P450 and oxidoreductase 2OG-FeII, both of which were reported to be upregulated in response to oxidative stress in *Arabidopsis*[20]. The protein phosphorylation category included several protein kinases, which may participate in signal transduction during many cellular processes under –P conditions, including metabolism, transcription, and cell cycle progression and differentiation. The lipid metabolic process category had many instances of lipase, which can alter membrane lipid composition to maintain internal Pi homeostasis under −P [21].

### Analysis of *IPS1* expression by RNA-Seq and qRT-PCR

Because RPKM values obtained from RNA-Seq indicated significant upregulation of *TaIPS1,* the wheat *IPS1* homologue, in root and shoot samples after 10 d of –P conditions, its expression levels were further analysed*. IPS1*, a non-protein coding gene that includes highly conserved sequences near miR399 complementary regions in different plant species [22], has been found to be strongly upregulated under −P in rice [10]. The homologous sequences *TaIPS1.1* (accession number EU753150), *TaIPS1.2* (EU753151), and *TaIPS1.3* (EU753152) have been isolated and deposited in GenBank, but molecular studies, such as expression analyses, have not been reported.

**Figure 4:**
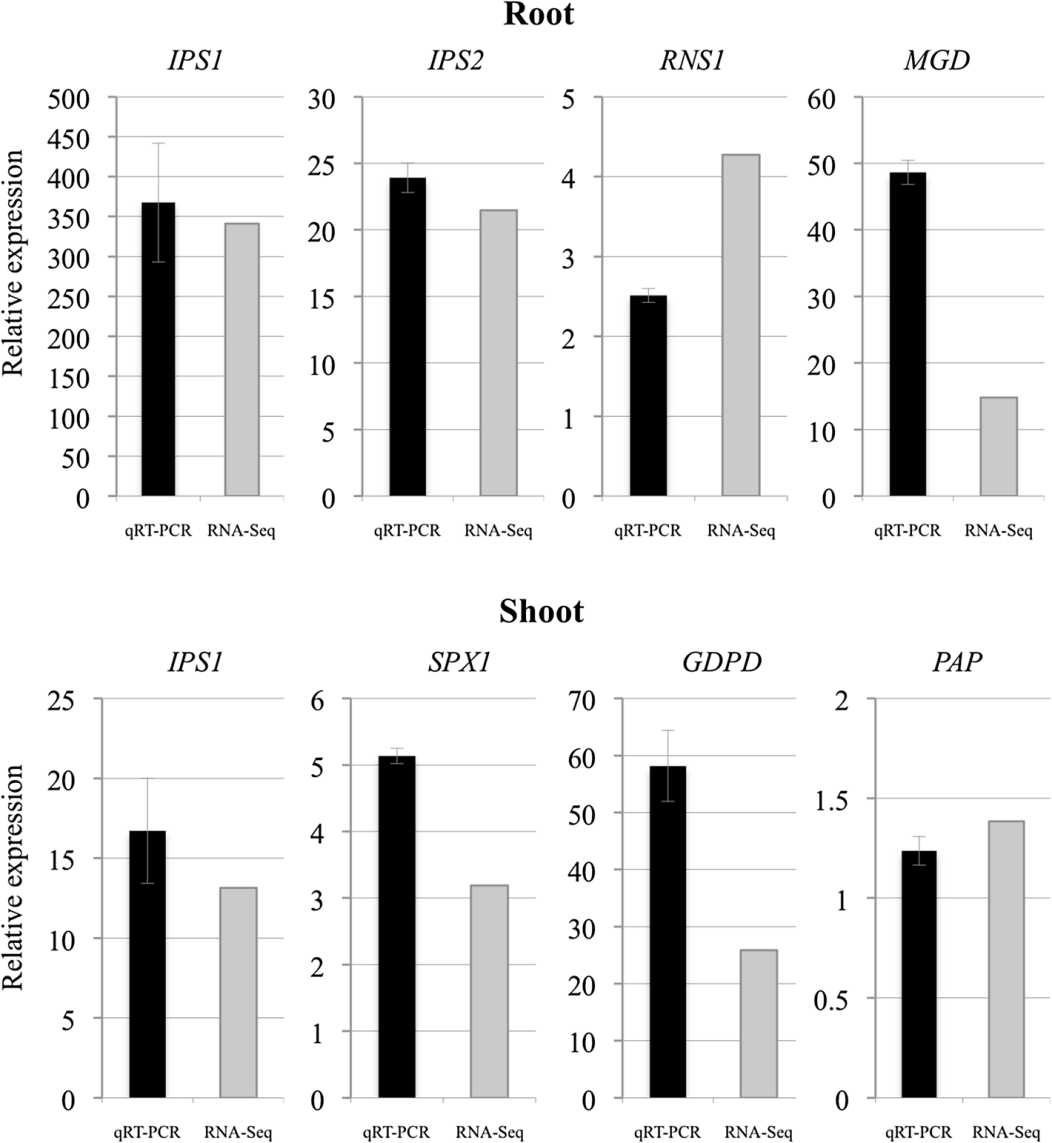
**Expression of *IPS1 *and several upregulated transcripts under –P conditions was determined using RNA-Seq and qRT-PCR.***IPS1*, *IPS2, RNS1*, and *MGD* expression in roots and *IPS1*, *SPX1, GDPD*, and *PAP* expression in shoots under –P were also analysed using qRT-PCR at 0 and 10 d after –P treatment. Transcript expression levels were normalised using an internal control (*Ubiquitin1*) and plotted relative to expression on day 10. Bars represent mean ± SE from the three experiments. Fold changes based on RPKM values according to RNA-Seq are plotted on the same graph.

Based on RNA-Seq, expression levels increased 341-fold in roots and 13-fold in shoots under –P conditions. In this study, we did not distinguish homeologous forms, and we present the results for each gene as an integration of counts from the three genomes of hexaploid wheat as reported by Pellny et al. [23]. The primary goal of our study to identify –P-responsive wheat genes, but we noted that using an integration of counts from the three homeologous forms for each gene could cause problems with transcript abundance when reads were aligned back to the wheat transcript set using Bowtie. A quantitative real-time PCR (qRT-PCR) analysis was used to confirm these results under identical conditions. Expression levels increased 368-fold in roots and 17-fold in shoots according to qRT-PCR, confirming the results obtained using RNA-Seq analysis (Figure 4). These data demonstrated that these transcriptomes accurately reflected the response of wheat to Pi starvation and suggest that the *IPS1*-mediated signalling cascade may also function in wheat.

### A highly conserved PHR1-IPS1-miR399-UBC24/PHO2 signalling cascade

Members of the *IPS1* gene family from different plant species, including rice, have a highly-conserved 23-nt-long motif that exhibits complementarity with the miR399 involved in Pi response [22]. Because *IPS1* is strongly upregulated in rice [10], we investigated the conserved responsive genes by comparing the wheat transcripts with rice transcripts used in our previous RNA-Seq study [10]. To assign Rice Annotation Project (RAP) IDs to responsive transcripts in wheat under –P conditions, we searched for homology to RAP (http://rapdb.dna.affrc.go.jp/) proteins using BLASTP. Of the 29,617 wheat transcripts in our constructed dataset, open reading frames (ORFs) were predicted for 21,864. Among these, 9,435 transcripts with reciprocal top hits (E-value ≤1E-06) were identified.

A total of 7,449 transcripts were found to be orthologous to their corresponding rice genes based on a threshold of ≥ 60% identity and ≥ 60% coverage. In addition, BLASTN was used to identify rice orthologues of wheat non-protein coding genes. Orthologues of the responsive wheat transcripts were verified for their responses by comparing their data with those of the responsive rice transcripts. Because most well-characterised transcripts have been found to be upregulated under −P conditions in plants, we focused on the upregulated transcripts (Figure 2). In wheat, 376 of the 1,004 upregulated transcripts in roots and 1,326 of the 2,833 upregulated transcripts in shoots possessed orthologues, which were then compared with those found in the responsive rice transcripts. Thirty-nine transcripts in roots and 21 transcripts in shoots were upregulated in both wheat and rice, suggesting their importance in adaptation to –P conditions in Poaceae species (Table 3). The 16 transcripts upregulated in both roots and shoots are of particular interest.

**Table 3:** Upregulated transcripts found in both wheat and rice under –P conditions

**Transcript ID**	**Source**	**Tissue**	**Length**	**RPKM (0d)**	**RPKM (10d)**	**Fold change**	**RAP transcripts**	**Gene**	**Gene annotation**	**P1BS in 1-kb upstream region of rice gene**
RFL_Contig1729	TriFLDB	Root	756	1.14	389.75	340.97	Os03t0146800-01	IPS1	Non-protein coding transcript	+
tplb0015j18	TriFLDB	Shoot	1,571	3.88	100.42	25.87	Os02t0514500-01	GDPD	Glycerophosphoryl diester phosphodiesterase family protein	-
RFL_Contig1771	TriFLDB	Root	391	6.30	135.25	21.46	Os01t0838350-01	IPS2	Conserved hypothetical protein	**+**
RFL_Contig2948	TriFLDB	Root	1,360	22.19	373.52	16.83	Os06t0603600-01	SPX1	SYG/PHO8/XPR1 (SPX) domain gene	-
tplb0011c17	TriFLDB	Shoot	1,685	2.29	37.19	16.22	Os08t0299400-01	MGD	MGDG synthase type A	-
tplb0011c17	TriFLDB	Root	1,685	2.02	29.80	14.78	Os08t0299400-01	MGD	MGDG synthase type A	-
RFL_Contig1729	TriFLDB	Shoot	756	8.71	114.37	13.13	Os03t0146800-01	IPS1	Non-protein coding transcript	**+**
RFL_Contig1771	TriFLDB	Shoot	391	1.81	20.83	11.51	Os01t0838350-01	IPS2	Non-protein coding transcript	**+**
tplb0009k23	TriFLDB	Root	1,609	6.46	53.39	8.26	Os02t0325600-01	-	Similar to phosphate starvation response regulator-like protein	**+**
tplb0015j18	TriFLDB	Root	1,571	21.91	170.15	7.77	Os02t0514500-01	GDPD	Glycerophosphoryl diester phosphodiesterase family protein	-
tplb0009k23	TriFLDB	Shoot	1,609	3.35	21.14	6.30	Os02t0325600-01	-	Similar to phosphate starvation response regulator-like protein	**+**
Contig.28393.5	Assembled	Shoot	1,075	1.17	6.69	5.73	Os08t0433200-01	-	Conserved hypothetical protein	**+**
tplb0017d11	TriFLDB	Root	2,344	2.12	10.98	5.18	Os04t0555300-01	-	Similar to glycerol 3-phosphate permease	**+**
Contig.28393.5	Assembled	Root	1,075	1.00	4.64	4.64	Os08t0433200-01	-	Conserved hypothetical protein	**+**
RFL_Contig180	TriFLDB	Root	929	151.92	649.17	4.27	Os07t0630400-01	OsRNS1	Ribonuclease T2 family protein	**+**
tplb0002b13	TriFLDB	Root	915	16.38	69.34	4.23	Os08t0434100-01	OsRNS3	S-like ribonuclease	**+**
Contig.8101.10	Assembled	Root	2,058	3.74	14.18	3.79	Os09t0321200-00	-	Similar to carotenoid cleavage dioxygenase	**+**
tplb0010d01	TriFLDB	Shoot	1,435	2.20	8.28	3.76	Os08t0535700-00	-	Similar to glycerophosphodiester phosphodiesterase	**+**
Contig.9738.4	Assembled	Root	2,060	7.56	27.93	3.70	Os06t0325200-00	Pht1;10	Major facilitator superfamily protein	-
tplb0005p16	TriFLDB	Root	2,900	25.71	91.49	3.56	Os10t0100500-01	-	Serine/threonine protein kinase-related domain containing protein	**+**
RFL_Contig2948	TriFLDB	Shoot	1,360	22.46	71.55	3.19	Os06t0603600-01	SPX1	SYG/PHO8/XPR1 (SPX) domain gene	-
Contig.7073.9	Assembled	Root	2,151	10.97	32.96	3.01	Os12t0554500-00	-	Lipase, class 3 family protein	-
tplb0004l11	TriFLDB	Root	1,241	86.40	210.59	2.44	Os04t0652700-01	-	Similar to nuclease PA3	-
Contig.19551.3	Assembled	Root	756	3.83	9.28	2.42	Os08t0478000-01	-	Similar to mucin-2	-
RFL_Contig6043	TriFLDB	Shoot	1,744	29.56	69.86	2.36	Os05t0475400-01	-	Similar to alanine:glyoxylate aminotransferase-like protein	-
Contig.7627.18	Assembled	Root	1,951	14.40	29.37	2.04	Os06t0115600-01	-	Similar to CYCLOPS	-
Contig.1419.6	Assembled	Root	845	12.66	24.57	1.94	Os09t0367700-01	-	Similar to GST6 protein	**+**
RFL_Contig5356	TriFLDB	Shoot	2,134	62.44	117.43	1.88	Os01t0557500-01	-	Cation/proton exchanger 1a	-
RFL_Contig1089	TriFLDB	Root	1,979	15.97	29.71	1.86	Os05t0387200-01	SQD1	Sulfite:UDP-glucose sulfotransferase	-
RFL_Contig3773	TriFLDB	Root	1,915	14.45	26.40	1.83	Os07t0100300-02	-	Glycosyl transferase, group 1 domain containing protein	**+**
Contig.5729.19	Assembled	Root	2,057	16.32	29.61	1.81	Os05t0489900-01	-	Calcium/calmodulin-dependent protein kinase	-
tplb0005j01	TriFLDB	Shoot	1,911	42.84	71.03	1.66	Os03t0760200-01	-	Cytochrome P450 family protein	**+**
RFL_Contig3516	TriFLDB	Root	2,119	158.24	256.48	1.62	Os05t0137400-01	-	Similar to aspartic protease precursor	**+**
RFL_Contig1089	TriFLDB	Shoot	1,979	103.87	166.66	1.60	Os05t0387200-01	SQD1	Sulfite:UDP-glucose sulfotransferase	-
RFL_Contig3516	TriFLDB	Shoot	2,119	123.51	190.78	1.54	Os05t0137400-01	-	Similar to aspartic protease precursor	**+**
RFL_Contig3173	TriFLDB	Root	2,096	93.45	143.24	1.53	Os03t0826600-01	-	Similar to phospholipase	**+**
tplb0011n16	TriFLDB	Root	3,325	506.03	752.10	1.49	Os09t0315700-01	-	Phosphoenolpyruvate carboxylase family protein	-
RFL_Contig3773	TriFLDB	Shoot	1,915	119.10	170.78	1.43	Os07t0100300-02	-	Glycosyl transferase, group 1 domain containing protein	**+**
tplb0011n16	TriFLDB	Shoot	3,325	122.46	174.90	1.43	Os09t0315700-01	-	Phosphoenolpyruvate carboxylase family protein	-
tplb0006o12	TriFLDB	Root	1,917	13.84	19.77	1.43	Os01t0109300-01	-	Similar to predicted protein	**+**
tplb0002m14	TriFLDB	Root	1,205	156.19	221.81	1.42	Os01t0897200-04	OsRNS2	Ribonuclease 2 precursor	**+**
RFL_Contig3173	TriFLDB	Shoot	2,096	93.61	132.02	1.41	Os03t0826600-01	-	Similar to phospholipase	**+**
RFL_Contig2349	TriFLDB	Root	3,073	304.03	425.90	1.40	Os02t0809800-01	PHO1:H2	Root-to-shoot inorganic phosphate (Pi) transfer	-
RFL_Contig3543	TriFLDB	Shoot	1,308	187.76	259.91	1.38	Os03t0238600-01	PAP	Similar to purple acid phosphatase	-
RFL_Contig5356	TriFLDB	Root	2,134	20.40	28.11	1.38	Os01t0557500-01	-	Cation/proton exchanger 1a	-
tplb0013h02	TriFLDB	Root	2,851	62.68	83.60	1.33	Os10t0500600-01	-	Zinc finger, C2H2-like domain containing protein	**+**
RFL_Contig5307	TriFLDB	Root	1,219	43.36	57.80	1.33	Os01t0128200-01	-	Similar to nuclease I	-
tplb0004m05	TriFLDB	Root	942	42.30	55.89	1.32	Os02t0704900-02	-	Similar to inorganic pyrophosphatase-like protein	**+**
tplb0011n23	TriFLDB	Root	2,648	105.24	137.60	1.31	Os01t0621900-02	-	Conserved hypothetical protein	-
tplb0008m02	TriFLDB	Root	1,657	149.03	190.13	1.28	Os08t0557600-01	-	Similar to monodehydroascorbate reductase	-
RFL_Contig3543	TriFLDB	Root	1,308	597.17	755.77	1.27	Os03t0238600-01	PAP	Similar to purple acid phosphatase	-
RFL_Contig6043	TriFLDB	Root	1,744	272.35	333.30	1.22	Os05t0475400-01	-	Similar to alanine:glyoxylate aminotransferase-like protein	-
tplb0001a12	TriFLDB	Shoot	1,678	23.44	28.13	1.20	Os06t0204400-01	-	Similar to aminoalcoholphosphotransferase	-
tplb0010b10	TriFLDB	Root	1,838	295.07	343.13	1.16	Os09t0553200-01	UGPase	UDP-glucose pyrophosphorylase	-
tplb0017h23	TriFLDB	Shoot	1,988	97.39	112.72	1.16	Os09t0478300-01	-	Conserved hypothetical protein	-
RFL_Contig3605	TriFLDB	Root	2,532	72.22	83.43	1.16	Os09t0379900-02	-	-	-
tplb0004a13	TriFLDB	Shoot	2,730	389.19	449.07	1.15	Os06t0178900-01	-	Vacuolar H + −pyrophosphatase	-
tplb0010b10	TriFLDB	Shoot	1,838	338.48	380.45	1.12	Os09t0553200-01	UGPase	UDP-glucose pyrophosphorylase	-
tplb0006p01	TriFLDB	Root	1,330	1139.67	1233.89	1.08	Os08t0126300-02	-	Similar to glyceraldehyde-3-phosphate dehydrogenase	-
tplb0013o11	TriFLDB	Root	2,022	197.87	210.17	1.06	Os07t0622200-01	-	Similar to M-160-u1_1	-

**Figure 5:**
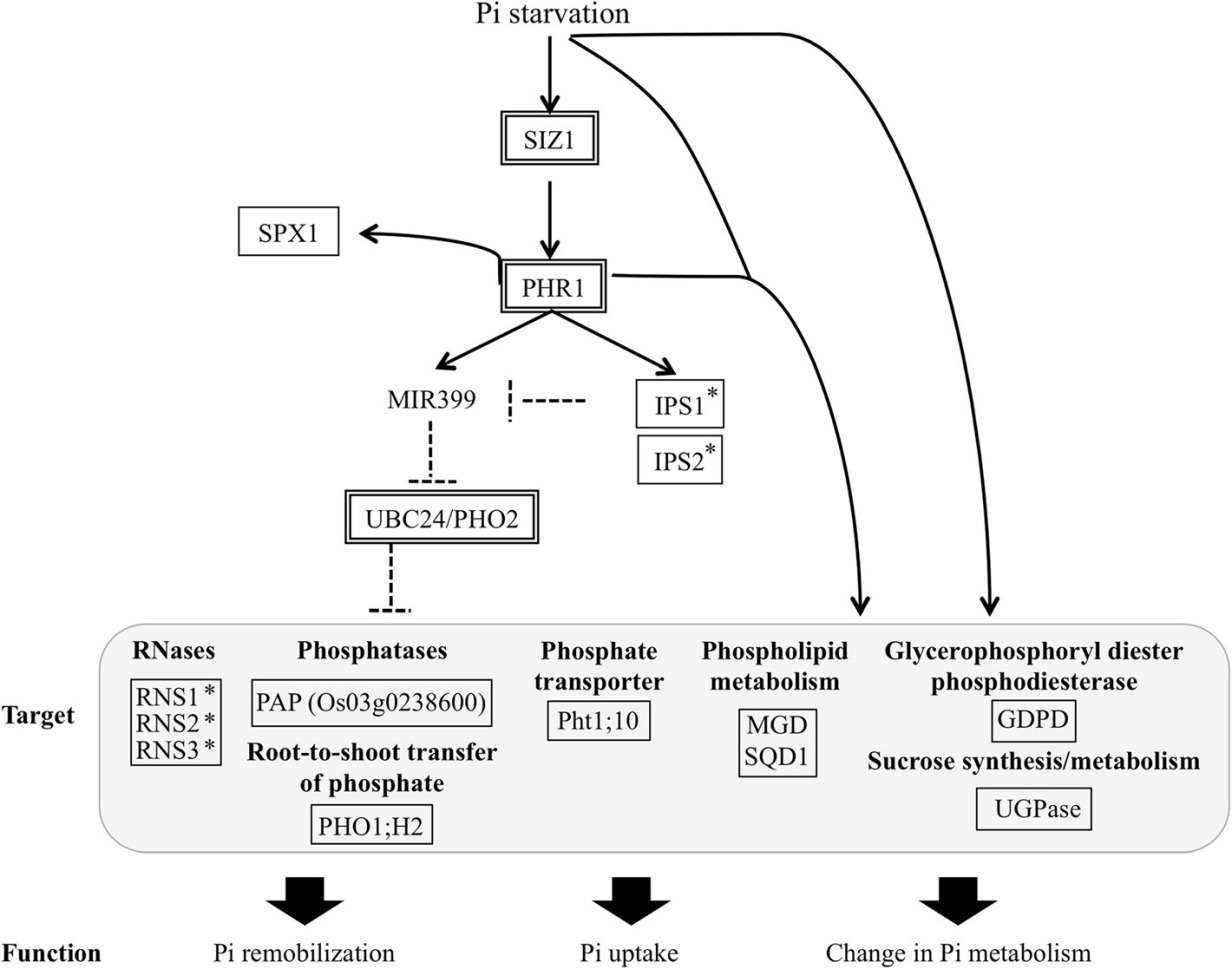
**Overview of the P-dependent signalling cascades that affect Pi remobilisation, Pi uptake, and changes in Pi metabolism.** The functions of wheat transcripts and rice orthologues of Pi-related transcripts were characterised under –P conditions. Asterisks show that the P1BS *cis*-acting element (GNATATNC), an imperfect palindromic sequence [24], is located in the upstream region (1 kb).

The Pi regulatory mechanism has been elucidated through *Arabidopsis* mutant analyses and involves PHR1 (phosphate starvation response 1), a MYB-type transcription factor, acting as a key factor in regulating downstream –P-responsive gene expression, including that of *IPS1,* through the P1BS *cis*-acting element (GNATATNC) of the PHR1 binding site (Figure 5). This mechanism is conserved in vascular plants and unicellular algae [24]. As in conserved genes in wheat and rice, the P1BS *cis*-element exists in the promoter of wheat-orthologous rice genes; *IPS1* is upregulated primarily in the roots of wheat (Table 3) and rice [10], suggesting conservation of the *IPS1*-mediated signalling cascade under the control of PHR1 (PHR2 in rice [25]). In *Arabidopsis*, SIZ1 (small ubiquitin-like modifier [SUMO] E3 ligase) [16], miR399, and UBC24/PHO2 (ubiquitin E2 conjugase) are also involved in the cascade (Figure 5) [26]. According to genome sequencing information, SIZ1 is conserved in rice [27]; miR399 expression has been confirmed in rice, and a potential orthologue of UBC24/PHO2 has been identified by assembly using genomic DNA and expressed sequence tags (ESTs) [26]. Transcribed sequences encoding miR399, miR399-binding sites, or protein sequences homologous to N- or C-terminal extensions of UBC24/PHO2 have been observed in wheat [26]. We also detected rice-orthologous wheat transcripts of *PHR1*, *UBC24/PHO2*, and *SIZ1*, although these transcripts were not highly responsive to −P in wheat (data not shown) or rice [10]. Mature miRNAs shorter than the sequencing length of the short reads could be difficult to detect in our RNA-Seq libraries, however. Based on the identification of HvmiR399s [28] and the upregulation of *HvIPS1*[29], the IPS1-miR399-UBC24/PHO2 signalling cascade appears to be conserved in barley [18]. Consequently, the PHR1-IPS1-miR399-UBC24/PHO2 signalling cascade is probably also conserved in wheat (Figure 5). *In silico* comparative analysis [30] suggested the presence of conserved *cis*-acting elements, such as the P1BS-like motif, and *trans*-acting factors that are capable of regulating the sole putative wheat high-affinity phosphate transporter TaPT2, which is expressed in a tissue-specific and Pi-dependent fashion in both monocots and dicots.

We also studied the upregulated expressions of other responsive rice-orthologous wheat transcripts (Table 3). *SPX1,* upregulated under –P conditions, acts as a negative regulator for the expression of *PHR2* and, consequently, Pi transporters in rice [31]. RNS1-3 (RNase) [32], PAP (purple acid phosphatase) [33], and PHO1 (which transfers Pi from roots to shoots in rice [34]) may function in the remobilisation of Pi. In *Arabidopsis*, PHR1 SUMOylated by SIZ1 positively regulates *RNS1* expression [35]. Pht1;10 (phosphate transporter) [36] may function in the uptake of Pi. *AtPht1;8* repression and *AtPht1;9* expression are attenuated in the *pho2* mutant of *Arabidopsis*[26].

Genes involved in lipid metabolism, such as those that function in the synthesis of galactolipids and sulfolipids, were strongly induced under −P conditions. These include SQD1 (UDP-sulfoquinovose synthase 1) [37], MGD (MGDG [monogalactosyldiacylglycerol] synthases) [38], and GDPD (glycerophosphoryl diester phosphodiesterase) [39], which may work to re-route Pi during lipid metabolism. UDP-glucose pyrophosphorylase may act as a sugar-signalling network under –P conditions [40,41]. These factors may be related to the PHR1-IPS1-miR399-UBC24/PHO2 signalling cascade, as Pi remobilisation, Pi uptake, and Pi-related metabolism are under the control of that cascade (Figure 5). Upregulation of several transcripts in this cascade was also confirmed by qRT-PCR (Figure 4). These results suggested that the PHR1-IPS1-miR399-UBC24/PHO2 signalling cascade functions as a potent adaptation to −P in wheat.

### Transcriptional control of other −P responsive transcripts

We identified several upregulated regulatory wheat transcripts involved in −P response from top hits to *Arabidopsis* proteins using BLASTP. (Contig transcript searches were performed with BLASTX.) *WRKY6 (RFL_Contig5159)* and *PHO1 (RFL_Contig2349)* transcripts were upregulated in roots. In *Arabidopsis*, enhanced *WRKY6* mutants are more sensitive to −P conditions, having lower Pi contents in shoots compared with wild-type seedlings [42]. WRKY6 is involved in responses to –P by regulating *PHOSPHATE1 (PHO1)* expression [42]. PHO1 plays a role in Pi translation from roots to shoots, aiding plant adaptation to –P [43]. Salt and mannitol stress-inducible *WRKY33 (tplb0001n04)* and *SKIP (tplb0006k10)*[44,45], and *WRKY70* (*Contig.7004.23*) [46], which have pivotal roles in determining the balance between salicylic acid- and jasmonic acid-dependent defence pathways, were also upregulated in shoots. *SWI3C (RFL_Contig4840), SWI3D (tplb0017g02)*, and *GCN5-related N-acetyltransferase (Contig.1767.6)* were upregulated in shoots. SWI/SNF (SWITCH/SUCROSE NONFERMENTING) chromatin-remodelling complexes mediate ATP-dependent alterations of DNA-histone contacts. Histone H2A.Z regulates the expression of several classes of phosphate starvation response genes [47]. Although mechanisms of transcription rate modulation entailing chromatin structure alteration have not been fully elucidated under –P in *Arabidopsis*, SWI/SNF complexes and Gcn5 histone acetyltransferase are necessary for full induction of several phosphatase genes and PHO84 high-affinity phosphate transporter gene in yeast [48-52].

Finally, we investigated downregulated wheat transcripts under –P and found that *PsbQ-like 1 (RFL_Contig3541), PsbQ-like 2 (RFL_Contig550), PSBP-1 (tplb0004p24), PSBQ-1 (tplb0011f13), LHCB1.5 (tplb0016n06), LHCA6 (RFL_Contig363), LHCB3 (tplb0013f07)*, and *Photosystem I reaction centre subunit N (tplb0007a14)* were downregulated in shoots. These genes are involved in photosynthesis. In *Brassica nigra* leaf petiole suspension cells, the rate of photosynthesis and photosynthetic product partitioning were altered under –P [53]. *Glyceraldehyde-3-phosphate dehydrogenase (RFL_Contig1308)* and *sedoheptulose-1,7-bisphosphatase (SBPase) (tplb0007f04)* were also downregulated in shoots under –P. These are key regulatory enzymes in CO_2_ reduction and the regeneration phase of the Calvin cycle for carbon fixation pathways, respectively. SBPase, which catalyses the dephosphorylation of sedoheptulose-1,7-bisphosphate into sedoheptulose-7-phosphate and Pi, is specific to the eukaryotic Calvin cycle and plays vital roles in regulating the pathways in the cycle [54] and improving photosynthetic capacity [55]. Downregulation under –P of transcripts associated with photosynthesis and alteration of the balance of carbon metabolism are well documented in *Arabidopsis*[8].

## Conclusions

In this study, we demonstrated the use of short-read sequence data to rapidly characterise a wheat transcriptome and have contributed significantly to the corpus of wheat transcript data. Differentially-expressed transcripts under –P in wheat included, on average, 23.4% of the contigs not covered by cDNAs in TriFLDB. The induction of these responses requires a sophisticated regulatory system, however, and details of this regulation in wheat have only recently begun to be elucidated. Comparison of upregulated wheat and rice transcripts revealed that the PHR1-IPS1-miR399-UBC24/PHO2 signalling cascade is conserved in both crop species. Data from our previous study of rice transcripts and other supportive studies in other species confirmed that our analysis captured the transcriptome for –P response in wheat. This study thus represents a genomic approach to discovering wheat transcripts when genome sequences are unavailable. This contribution is significant to the development of genomic resources for wheat and other species and should accelerate the progress of functional genomic studies and breeding programs.

## Methods

### Plant materials and stress treatment

Seeds of the wheat cultivar ‘Chinese Spring’ were germinated and grown hydroponically in nutrient media (0.323 mM NaH_2_PO_4_; 10 mg L^−1^ P) [56] in a growth chamber. After 14 d, seedlings were subjected to a –P stress treatment by being transferred to a similar medium (0 mM NaH_2_PO_4_; 0 mg L^−1^ P). Plants were maintained under –P for 10 d at 23°C under a 16 h light/8 h dark cycle, with the light period extending from 6:00 AM to 10:00 PM. Root and shoot samples were collected as described previously [10].

### RNA isolation and quality control

Total RNA was extracted from all tissue samples using a RNeasy Plant Mini kit (Qiagen, Hilden, Germany). RNA quality was assessed using a Bioanalyzer (Agilent, Palo Alto, CA, USA); as suggested by the manufacturer, only those samples with RIN (RNA integrity number) scores greater than 8.0 were used in subsequent analyses.

### Illumina sequencing and quality control

Twelve paired-end (PE) cDNA libraries were used to generate 107,298,935 PE reads from root and shoot tissues. Sequencing was performed on each library to generate 100-bp PE reads for transcriptome sequencing on an Illumina High-Seq 2000 platform. Library construction was accomplished using commercial products (Illumina), with sequencing performed by commercial service providers (Takara, Shiga, Japan). The sequence data generated in this study were deposited in the DDBJ Sequence Read Archive (Accession No. DRA000737). Low-quality bases (Q < 15) were trimmed from both ends using a customised program, and the adapters were trimmed using Cutadapt ver. 1.0 (http://code.google.com/p/cutadapt/) with the parameters ‘-f fastq -e 0.1 -O 5 -m 20’.

### *De novo* assembly

To generate a non-redundant set of transcripts, we performed a *de novo* assembly with publicly available programs using 73,804,720 PE RNA-Seq reads (36,902,360 pairs; 97.5 average read length, 7,198,965,163 total bases, 260 average insert length) obtained from four libraries, with each library consisting of three replicates of one of the four tissue/treatment combinations. Reads from each library were aligned to cDNAs in TriFLDB using Bowtie, and the calculated RPKM values were used to conduct a correlation analysis of the replicates. The replicate pair with the highest correlation coefficient among the three replicate pairs was selected for *de novo* assembly. Trans-ABySS (ver. 1.3.2) [16], Velvet (ver. 1.2.03; http://www.ebi.ac.uk/~zerbino/velvet/)-Oases (ver. 0.2.05; http://www.ebi.ac.uk/~zerbino/oases/) [17], and Trinity (ver. r2012-01-25, --seqType fq and --kmer_method meryl) [18] were used in this study. Those programs were developed to assemble short reads using a de Bruijn graph algorithm [57]. Various assembly parameters were also optimised to obtain the best results from each program. Trinity, a program developed specifically for *de novo* transcriptome assembly from short-read RNA-Seq data, was also used to assemble the single *k*-mers (k = 21). Trinity has been shown to be efficient and sensitive in recovering full-length transcripts and isoforms in yeast, mice, and whiteflies [18]. Velvet (‘-fastq –short’ [velveth], ‘-read_trkg yes’ [velvetg])-Oases (default parameters) and ABySS (ver. 1.3.3, OVERLAP_OPTIONS=‘--no-scaffold’ SIMPLEGRAPH_OPTIONS=‘--no-scaffold’) [58] were also used to assemble individual *k*-mer lengths from every odd number between 21 and 51.

Using a multiple *k*-mer (MK) strategy for the *de novo* assembly gave better results than did a non-multiple *k*-mer approach because it was able to capture both weakly expressed transcripts with small *k*-mer values and highly expressed genes with large *k*-mer values [59]. In this study, Velvet-Oases (‘-merge yes’) and Trans-ABySS (default) were used to assemble different *k*-mer lengths from 21 to 51; both yielded better results than those obtained with the individual *k*-mer approach. We therefore used the MK strategy for downstream analyses. We also performed *de novo* assembly with Oases and Trans-ABySS at different *k*-mer lengths, using every odd number from 21 to 95; these results did not improve the assessment. To remove redundant contigs, short contigs that were entirely covered by longer ones with more than 90% identity were removed using cd-hit-est (ver. 4.5.7) [60] with default parameters. Furthermore, Oases produces isoforms from a locus; the longest transcripts of each locus were selected to construct a non-redundant set of contigs to identify responsive transcripts.

### Calculation of RPKM values and the detection of differentially-expressed transcripts

The PE RNA-Seq reads from each treatment were aligned back to the cDNA, and the transcripts were assembled using Bowtie 2 ver. 2.0.0-beta6, with the parameters ‘-k 2 --no-discordant --no-mixed -q’. Expression levels of all unique transcripts mapped onto the full-length cDNAs and contigs were quantified using the RPKM values [61]. The RPKM value of each transcript was calculated using uniquely-aligned reads with an in-house program; each value was incremented by 1 to avoid division by 0 following the fold change calculations. Differentially expressed transcripts were detected using a G-test with an FDR value cut-off < 0.01. Details of this process have been described in previous work [10].

### Gene ontology analysis

The GO terms assigned to the responsive transcripts were obtained from InterProScan 5 Release Candidate 2 (http://code.google.com/p/interproscan/) with the parameters ‘-f tsv -t p -dp –goterms’ for each GO category after those transcripts were predicted with ORFs using transcripts_to_best_scoring_ORFs.pl with the parameter ‘-B’ in Trinity (ver. r2012-04-27).

### qRT-PCR analysis of wheat transcripts

First-strand cDNA was synthesised using a Transcriptor First Strand synthesis kit (Roche, Basel, Switzerland). Expression of Pi-upregulated transcripts was analysed by qRT-PCR in a LightCycler 480 system (Roche) with transcript-specific primers (Additional file 6). The detection threshold cycle of each reaction was normalised using *Ubiquitin1* primers 5^′^-GGAGGACACAGAAAGGCAAC-3^′^ and 5^′^-CTCCGTGGTGGCCAGTAAGT-3^′^. Three technical replicates of each treatment were used in the analysis.

## Competing interests

The authors declare that they have no competing interests.

## Authors’ contributions

YO conceived and coordinated this study, performed the experiments, analysed and interpreted the data, and drafted the manuscript. FK, YK, and TY performed the general statistical analysis on the RNA-Seq data and participated in interpreting the results and in useful discussions. HH, TI, and TM provided valuable insights in the discussion and revision of the manuscript. All authors have read and approved the final manuscript.

## Supplementary Material

Additional file 1 **Comparison of the *de novo*assembly of three datasets using Trinity (*k*= 21), Oases (MK) and Trans-ABySS (MK) programs.** Bars indicate the number of contigs. The dashed line indicates the mean contig length, and the solid line indicates the median contig length. Click here for file

Additional file 2 Statistical analysis of the non-redundant set of wheat transcripts obtained from three assembly programs. Click here for file

Additional file 3 **Assessment of contigs aligned to full-length wheat cDNAs in TriFLDB.** All quality-controlled reads were aligned to these full-length cDNAs using Bowtie and three assembly programs. The proportion of numbers and bases of the full-length transcripts covered by each assembly program were calculated. Click here for file

Additional file 4 **Conservation of sequences between wheat transcripts and other land plants.** The number of contigs showing significant similarities (E-value <1E-03) when compared with nr according to BLASTX is shown. Click here for file

Additional file 5 **RPKM values of contigs and cDNAs in TriFLDB.** (PDF 77 kb) Click here for file

Additional file 6 PCR primers used for qRT-PCR analysis. Click here for file
