# Supplementary figures and images for: Characterisation of the wheat (triticum aestivum L.) transcriptome by de novo assembly for the discovery of phosphate starvation-responsive genes: gene expression in Pi-stressed wheat
Source: BMC Genomics. 2013 Feb 4;14:77. doi: 10.1186/1471-2164-14-77 (PMC3598684; doi:10.1186/1471-2164-14-77)

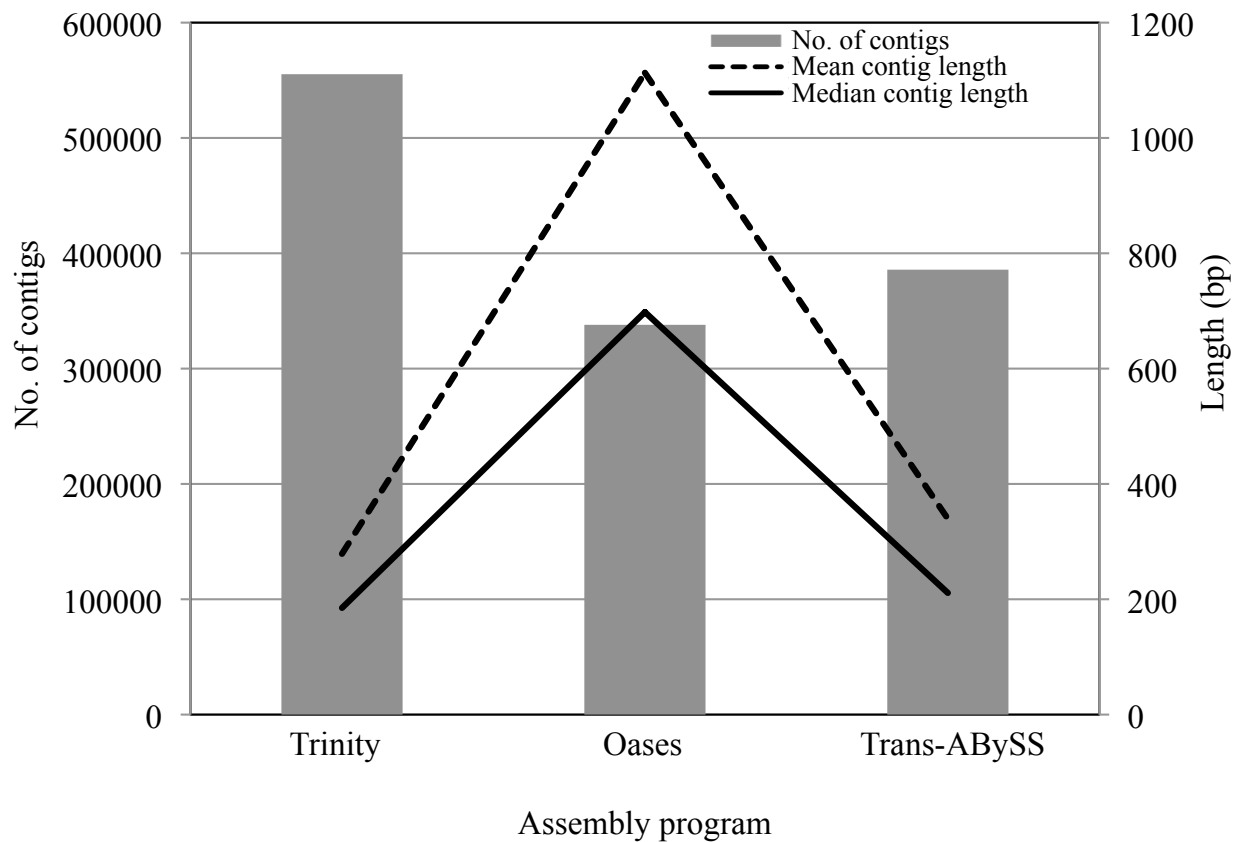

Supplement: Additional file 1 — Comparison of the de novoassembly of three datasets using Trinity (k= 21), Oases (MK) and Trans-ABySS (MK) programs. Bars indicate the number of contigs. The dashed line indicates the mean contig length, and the solid line indicates the median contig length. [file 1471-2164-14-77-S1.pdf]

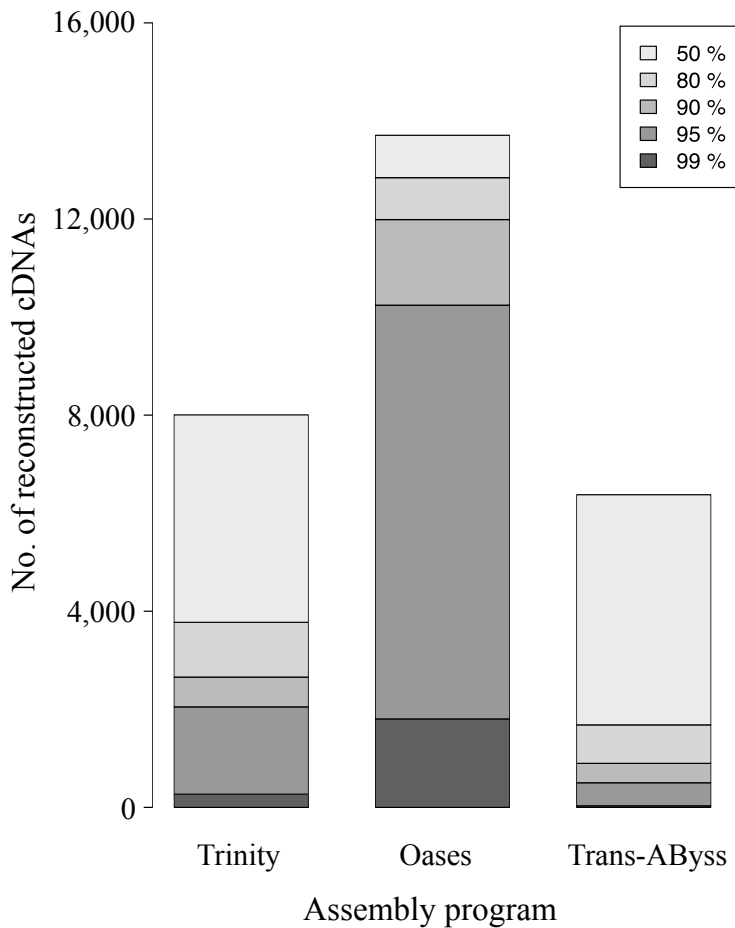

Supplement: Additional file 3 — Assessment of contigs aligned to full-length wheat cDNAs in TriFLDB. All quality-controlled reads were aligned to these full-length cDNAs using Bowtie and three assembly programs. The proportion of numbers and bases of the full-length transcripts covered by each assembly program were calculated. [file 1471-2164-14-77-S3.pdf]

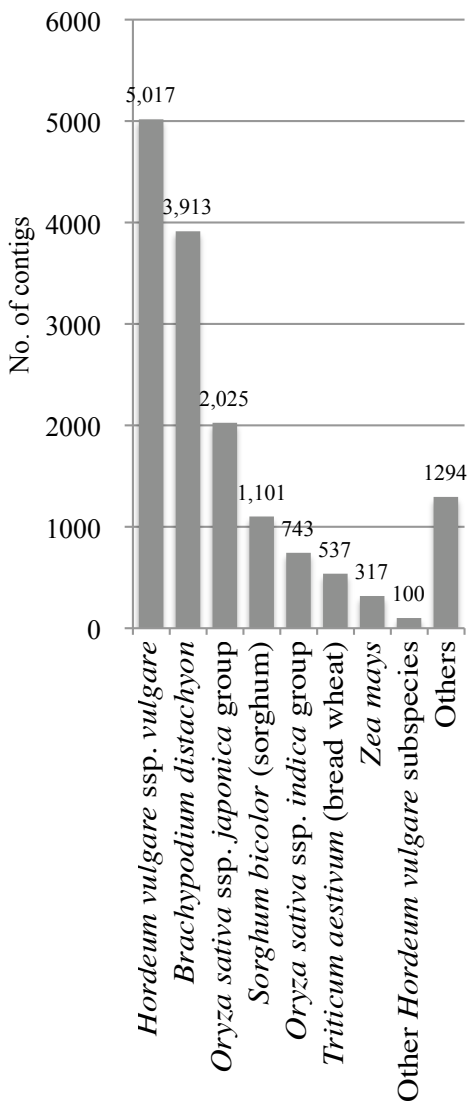

Supplement: Additional file 4 — Conservation of sequences between wheat transcripts and other land plants. The number of contigs showing significant similarities (E-value <1E-03) when compared with nr according to BLASTX is shown. [file 1471-2164-14-77-S4.pdf]
