# Supplementary material for: Characterisation of the wheat (triticum aestivum L.) transcriptome by de novo assembly for the discovery of phosphate starvation-responsive genes: gene expression in Pi-stressed wheat
Source: BMC Genomics. 2013 Feb 4;14:77. doi: 10.1186/1471-2164-14-77 (PMC3598684; doi:10.1186/1471-2164-14-77)
Supplement: Additional file 2 — Statistical analysis of the non-redundant set of wheat transcripts obtained from three assembly programs. [file 1471-2164-14-77-S2.pdf]

Additional File 2. Statistical analysis of the non-redundant set of wheat transcripts obtained from three assembly programs.

Assembly program	No. of contigs	Max. length	Min. length	Mean length	Median length
Trinity	555,287	15,020	101	279	185
Oases	337,969	22,610	100	1,113	698
Trans-ABySS	385,779	11,790	100	340	211
