# Supplementary material for: Characterisation of the wheat (triticum aestivum L.) transcriptome by de novo assembly for the discovery of phosphate starvation-responsive genes: gene expression in Pi-stressed wheat
Source: BMC Genomics. 2013 Feb 4;14:77. doi: 10.1186/1471-2164-14-77 (PMC3598684; doi:10.1186/1471-2164-14-77)
Supplement: Additional file 5 — RPKM values of contigs and cDNAs in TriFLDB. (PDF 77 kb) [file 1471-2164-14-77-S5.pdf]

Additional File 5. RPKM values of contig and cDNA in TriFLDB.

	Root_0d		Root_10d		Shoot_0d		Shoot_10d	
	Contig	cDNA	Contig	cDNA	Contig	cDNA	Contig	cDNA
Maximum RPKM	911.02	4191.02	1125.21	6085.74	643.79	8748.35	761.47	7524.68
Minimum RPKM	0.00	0.00	0.00	0.00	0.00	0.00	0.00	0.00
Average RPKM	4.44	31.95	4.75	32.01	2.75	33.61	2.93	33.68
Median RPKM	1.35	4.23	1.41	4.19	0.52	3.72	0.67	4.18
SD	14.72	107.16	16.93	122.61	12.05	193.32	11.89	163.74

Additional File 6. PCR primers for qRT-PCR analysis

Gene	PCR Primer 1	PCR Primer 2
IPS1	CGGCGACTTCTCACCTCTAC	GACACTGAAGACTCGCACCA
IPS2	TAGGCCGTGTAGGGCAACTA	GAGTTTCCACGAACGAGAGC
RNS1	AGCTGCCGGCCTTCTGATTA	TAATCCACCCGCGCACTGT
MGD	CGCGGAACCTCTTGCAGAAT	CTTCAGCAGGCATCCAAGC
SPX1	GACGCTGTGACACACGATAC	GAAACAGGTGAGGTCCTGGT
GDPD	GTGTGATCTCCTTCGGTGAG	AATCAATGCCCCCTCGCTAC
PAP	GATGCAGATCATGGCAGGAG	GCTGAACCACGCACCATACT
