# Supplementary material for: Characterisation of the wheat (triticum aestivum L.) transcriptome by de novo assembly for the discovery of phosphate starvation-responsive genes: gene expression in Pi-stressed wheat
Source: BMC Genomics. 2013 Feb 4;14:77. doi: 10.1186/1471-2164-14-77 (PMC3598684; doi:10.1186/1471-2164-14-77)
Supplement: Additional file 6 — PCR primers used for qRT-PCR analysis. [file 1471-2164-14-77-S6.pdf]

Additional File 6. PCR primers for qRT-PCR analysis

Gene	PCR Primer 1	PCR Primer 2
IPS1	CGGCGACTTCTCACCTCTAC	GACACTGAAGACTCGCACCA
IPS2	TAGGCCGTGTAGGGCAACTA	GAGTTTCCACGAACGAGAGC
RNS1	AGCTGCCGGCCTTCTGATTA	TAATCCACCCGCGCACTGT
MGD	CGCGGAACCTCTTGCAGAAT	CTTCAGCAGGCATCCAAGC
SPX1	GACGCTGTGACACACGATAC	GAAACAGGTGAGGTCCTGGT
GDPD	GTGTGATCTCCTTCGGTGAG	AATCAATGCCCCCTCGCTAC
PAP	GATGCAGATCATGGCAGGAG	GCTGAACCACGCACCATACT
